# A synthetic organelle approach to probe SNARE-mediated membrane fusion in a bacterial host

**DOI:** 10.1016/j.jbc.2023.102974

**Published:** 2023-02-03

**Authors:** Soledad Ferreras, Neha Pratap Singh, Remi Le Borgne, Philippe Bun, Thomas Binz, Robert G. Parton, Jean-Marc Verbavatz, Christian Vannier, Thierry Galli

**Affiliations:** 1 Université Paris Cité, Institute of Psychiatry and Neuroscience of Paris (IPNP), INSERM, Membrane Traffic in Healthy & Diseased Brain, Paris, France; 2 Université Paris Cité, CNRS, UMR7592, Institut Jacques Monod, Paris, France; 3 Université Paris Cité, NeurImag, Institute of Psychiatry and Neuroscience of Paris (IPNP), INSERM U1266, Paris, France; 4 Institute of Cell Biochemistry, Hannover Medical School, Hannover, Germany; 5 The University of Queensland, Institute for Molecular Bioscience and Centre for Microscopy and Microanalysis, Qld, Brisbane, Australia; 6 GHU Paris psychiatrie neurosciences, Paris, France

**Keywords:** SNARE proteins, synthetic biology, caveolin, membrane fusion, membrane reconstitution, AHT, anhydrotetracycline, BSA, bovine serum albumin, CNT, clostridial neurotoxin, MBP, maltose-binding protein, PBS-T, Triton in PBS, SNARE, soluble N-Ethylmaleimide–sensitive attachment protein receptor, TeNT-LC, light chain of tetanus neurotoxin

## Abstract

*In vivo* and *in vitro* assays, particularly reconstitution using artificial membranes, have established the role of synaptic soluble N-Ethylmaleimide–sensitive attachment protein receptors (SNAREs) VAMP2, Syntaxin-1A, and SNAP-25 in membrane fusion. However, using artificial membranes requires challenging protein purifications that could be avoided in a cell-based assay. Here, we developed a synthetic biological approach based on the generation of membrane cisternae by the integral membrane protein Caveolin in *Escherichia coli* and coexpression of SNAREs. Syntaxin-1A/SNAP-25/VAMP-2 complexes were formed and regulated by SNARE partner protein Munc-18a in the presence of Caveolin. Additionally, Syntaxin-1A/SNAP-25/VAMP-2 synthesis provoked increased length of *E. coli* only in the presence of Caveolin. We found that cell elongation required SNAP-25 and was inhibited by tetanus neurotoxin. This elongation was not a result of cell division arrest. Furthermore, electron and super-resolution microscopies showed that synaptic SNAREs and Caveolin coexpression led to the partial loss of the cisternae, suggesting their fusion with the plasma membrane. In summary, we propose that this assay reconstitutes membrane fusion in a simple organism with an easy-to-observe phenotype and is amenable to structure-function studies of SNAREs.

The question as to how eukaryotic cells establish spatial organization and control their specific dynamics of organelles in time and space is central in cell biology. The last 3 decades have seen the development of various approaches linking biochemistry, molecular biology, and genetics and aiming at reconstructing essential cellular functions (1, 2, 3). The ‘bottom-up’ synthetic approach aims to assemble biological systems with various degrees of complexity and exploits new powerful imaging and membrane reconstitution technologies (4, 5, 6). Here, we aimed to apply a synthetic biological approach to intracellular membrane fusion.

Soluble N-Ethylmaleimide–sensitive attachment receptors (SNAREs) constitute the core nanomachinery of intracellular membrane fusion. This was demonstrated by a large range of approaches principally using biochemistry and genetics (7). Vital evidence for the crucial role of synaptic SNAREs in mediating membrane fusion was provided by the observation that clostridial neurotoxins (CNTs), the most potent blockers of neurotransmitter release, are proteases that cleave the vesicular SNARE VAMP-2 or the target SNAREs Syntaxin-1A and SNAP-25 (8, 9). In particular, the light chain of tetanus neurotoxin (TeNT-LC) cleaves VAMP-2 and blocks neurotransmission (10). One of the demonstrations that SNAREs are sufficient for membrane fusion has been obtained by the expression of flipped v- and t-SNAREs at the surface of mammalian cells (11). SNARE studies have extensively relied on reconstitution assays with artificial membranes. *In vitro* or cell-free assays currently couple liposomes or suspended bilayers technologies and molecules obtained from cells or from synthesis with FRET and/or fluorescence microscopy (12, 13, 14). Consistently in such *in vitro* assays, the presence of VAMP-2 on one set of membrane and Syntaxin-1A and SNAP-25 on the other is necessary and sufficient for the membranes to fuse. *In vitro* assays further demonstrated that a limited number (1, 2, 3, 4, 5) of SNARE complexes were sufficient for efficient fusion (15, 16, 17). They confirmed the inhibitory role of cleavage by CNTs, allowed for structure-function studies, and the determination of biophysical parameters of membrane fusion (18). However, these assays are experimentally demanding because they require the production and purification of the full-length proteins to be studied and their insertion into artificial membranes. As cell-free reconstruction models rely on purified recombinant proteins and formation of multi-subunit complexes, stability of the protein during the course of expression and purification is a frequent issue, and several factors involved in both compositional and conformational stabilities have been identified (19). Bacteria may therefore represent a more stabilizing environment than *in vitro* assays to dissect complex cellular functions, such as signaling and metabolic pathways (20, 21, 22) or assembly and dynamics of supramolecular structures (23, 24). Recently, the biosynthetic pathway for major phosphoinositides was engineered into *Escherichia coli*, to circumvent their diverse functions and alternate synthesis pathways encountered in mammalian cells (25). *In vitro* membrane fusion assays also require complex microscopy and/or spectroscopic measurements with sensitive equipment, while cell-based assays are prone to the generation of a cellular morphological phenotype. Here, our goal was to develop a cell-based assay in bacteria in which membrane cisternae would be artificially generated and to test for the effect of expressing SNAREs, SNARE partner Munc-18a, and TeNT-LC.

Remarkably, this is now possible with the expression of a single protein in *E. coli* (26). In transformed cells intended for mammalian caveolin-1 (Cav) purification, the host produced heterologous caveolae derived from the plasma membrane in a fashion reminiscent of caveogenesis in mammalian cells. The formation of intracellular vesicles, accompanied by phospholipid segregation and a possible polyhedral arrangement of caveolin, is induced by WT human Cav but not noncaveogenic forms. Intriguingly, caveolin from *Caenorhabditis elegans* which was unable to generate caveolae in mammalian caveolin KO cells (27) was also unable to induce heterologous caveolae in *E. coli*. Instead, large intracytoplasmic membranous inclusions were generated by expansion of the plasma membrane. These inclusions likely resulted from the fact that *C. elegans* (Ce) Cav was not efficient in oligomerization and thus could not induce formation of free cytoplasmic vesicles. Such bacteria displaying intracytoplasmic cisternae provide a simple cellular system reconstituting the topology of the eukaryotic endomembrane system (28). A previous attempt to coexpress human caveolin and synaptic SNAREs was limited to the expression of VAMP-2 and Syntaxin-1A. This showed that the SNAREs had the correct topology and VAMP-2-bearing caveolae were able to fuse with Syntaxin-1A/SNAP-25 target liposomes *in vitro*. Altogether, this showed that VAMP-2 inserted in bacterial caveolae is functional *in vitro* (29). In theory, these cells display a potential intracellular membrane–delineated compartment that could be driven to a membrane fusion event by the additional synthesis of fusogenic proteins. This led us to undertake a full reconstitution of SNARE-mediated membrane fusion *in vivo* in bacteria. To this end, we expressed *CeCav* together with specific synaptic SNARE proteins and partners after selective induction from compatible expression plasmids (30), with the aim of mobilizing the cisternal membrane for fusion with the bacterial plasma membrane. We chose *CeCav* because it induces large cisternae (26) and we considered that intracellular fusion of such large membranes might be the best approach to obtain a striking phenotype. This idea set the basis for the assay shown here in which synaptic SNAREs and Cav are coexpressed in bacteria. Our goal was at the same time to test the hypothesis that basic membrane-trafficking reactions could be reconstituted in living prokaryotes with only a membrane vesiculating protein (Cav) and membrane fusion agents (SNAREs).

The formation of protein complexes consisting of Syntaxin-1A, SNAP-25, and VAMP-2 was regulated by coexpression of Munc-18a. Cav was shown to interact with Syntaxin 1. *E. coli*–synthesizing synaptic SNARE complexes exhibited an increased cell length, requiring presence of both Caveolin and SNAP-25, but prevented by TeNT-LC. Synaptic SNARE expression together with Cav was accompanied with a partial loss of the intracellular cisternae suggesting their fusion with the plasma membrane as revealed by electron and super-resolution microscopic analyses. Analysis of DNA content suggested that elongated cells did not suffer from mitosis arrest. These data can therefore be interpreted as the reconstitution of membrane fusion events in a simple cellular model in which using the simple morphological read-out of cell length, SNARE structure-function relationships can be analyzed.

## Results

**Figure 1:**
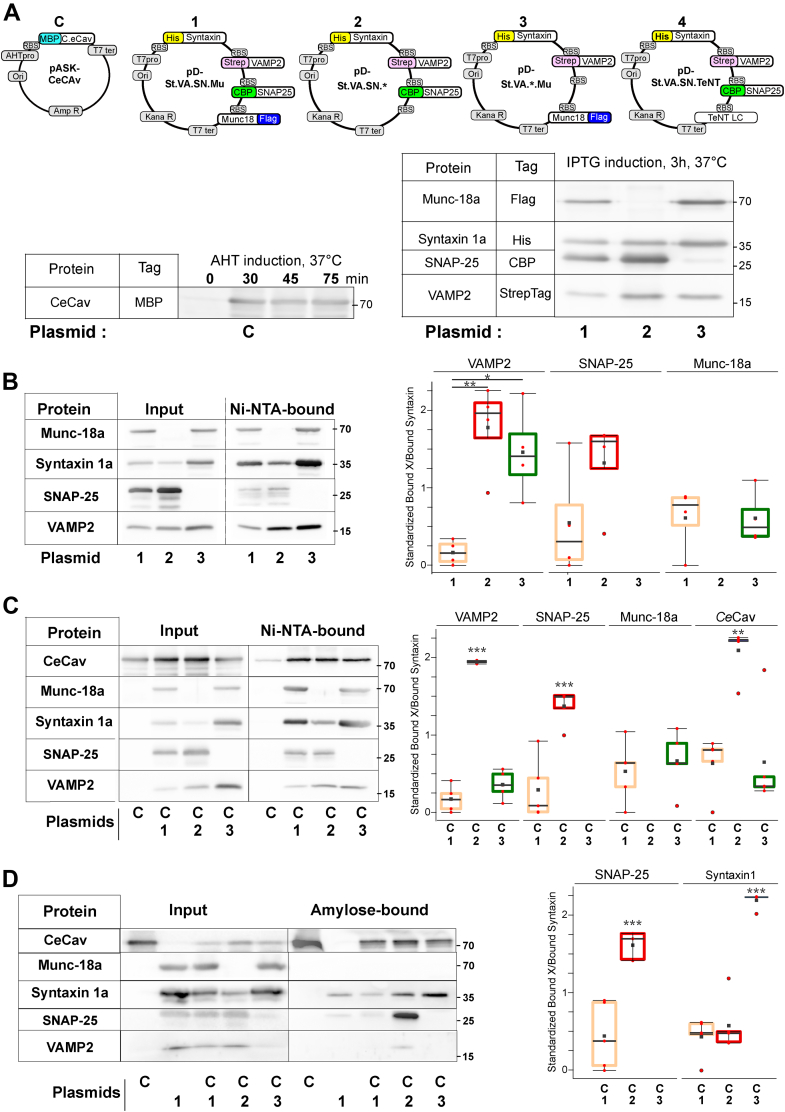
**A system to reconstitute synaptic SNAREs in Caveolin-expressing *Escherichia coli***. *A*, structure of recombinant plasmids driving expression, respectively, of *Ce* caveolin (pASK background), named plasmid C and of four combinations of SNARE (Syntaxin-1A, VAMP-2, SNAP-25) and accessory proteins (Munc-18a, TeNT), named plasmids 1 to 4 (modified pDuet background). The positions and nature of ORFs and DNA modules for antibiotic resistance, replication, and transcription/translation are depicted: RBS, ribosomal-binding site; Amp R and Kana R, ampicillin and kanamycin resistances; AHT pro, anhydro-tetracycline promotor; t7 pro, t promoter; t7 ter, t7 terminator; Ori, origin of replication. Leader sequences and tags are indicated: MBP, maltose-binding protein; Strep, Strep-tag; His, 6 histidine tag; CBP tag; Flag, flag-tag. Immunoblots of cell extracts showing synthesis of *CeCav* (*left panel*) and SNAREs proteins (*right panel*) upon expression from plasmid C or from plasmids 1 to 4 after AHT or IPTG induction, respectively. Reactive proteins were visualized by ECL. *B*–*D*, biochemical characterization of SNARE complexes present in *Escherichia coli* in the absence or presence of cytoplasmic-cisternae. Affinity chromatography on Ni-NTA matrix of soluble His_6_-syntaxin–containing complexes present in six A_600_-equivalent of bacterial cultures. Bacteria were transformed as indicated with one of the following plasmids: 1, 2, or 3, not combined (*B*) or combined (*C*) with plasmid C. Matrix-bound complexes were recovered in boiling Laemmli sample buffer and analyzed by SDS-PAGE and Western blotting using anti-syntaxin-1A, anti-Munc-18a, anti-VAMP-2, anti-SNAP-25, and anti-MBP antibodies. Affinity chromatography on amylose resin was performed using the same samples (D) to identify proteins associated with *CeCav*. Note that *CeCav* could capture Syntaxin-1A and/or SNAP-25 but not complexes containing VAMP-2 or Munc-18a. For ECL detection, in order to avoid signal saturation of probed proteins, only 1% of the starting material (Input) and 30% of the recovered bound material (Ni-NTA– or amylose-bound) were loaded on the same gel and transferred on the same membrane. Molecular weights are indicated (kDa). *Right panels* in (*B*–*D*) show the quantification of the indicated proteins in the matrix-bound material. ECL signals determined for the indicated proteins (X) by densitometry (using ImageJ software) and obtained from 4 to 5 independent experiments are plotted as Bound X/Bound Syntaxin (*B* and *C*) or Bound X/Bound *CeCav* after normalization. Each *p* value corresponds to the statistical one-way ANOVA (∗*p* ≤ 0.05; ∗∗*p* ≤ 0.01; ∗∗∗*p* ≤ 0.001.). *Red dots* in boxplots represent the standardized individual values of all experimental replicates and *black squares* correspond to the mean value of the data. SNARE, soluble N-Ethylmaleimide–sensitive attachment protein receptor.

In order to independently express a number of constructs including *CeCav*, synaptic SNAREs plus a potential partner or TeNT-LC in *E. coli*, two expression vectors were needed harboring two different replication origins, antibiotic resistances, and inducible transcription units. We took advantage of two backbones: pASK-IBA3 for the expression of *CeCav* (plasmid C) and modified pRSFDuet-1 for the expression of full-length Syntaxin-1A, SNAP-25, VAMP-2, and Munc-18a (plasmid 1); Syntaxin-1A, SNAP-25 and VAMP-2 (plasmid 2); Syntaxin-1A, VAMP-2 and Munc-18a (plasmid 3); or Syntaxin-1A, SNAP-25, VAMP-2, and TeNT-LC (plasmid 4). A different tag was fused to each individual protein (Fig. 1*A*). Cell culture and protein inductions were performed in chemically defined medium chosen to ensure low growth rate as well as preventing overexpression of exogenous proteins. We first checked the expression of the different proteins encoded in these vectors and found proper induction of *CeCav* by anhydrotetracycline (AHT) and synaptic SNAREs and Munc-18a by IPTG (Fig. 1*B*). We also confirmed that expression of *CeCav* generated intracellular cisternae as previously described. Cisternaes could be observed as early as 30 to 120 min after treatment with AHT at 37 °C and was very prominent after overnight induction with AHT at 25 °C (Fig. S1*A*).

We then checked the expression levels of the different proteins encoded in our vectors as a function of time of induction. In the absence of plasmid C, IPTG induced a strong and robust expression of Syntaxin-1A in the cases of plasmids 1, 2, and 3. In all cases, Syntaxin-1A was already expressed before IPTG induction. The same pattern of leaked expression and strong IPTG dependence was observed in the case of VAMP-2. Munc-18a was generally already expressed at its maximal level before addition of IPTG when cells were transformed with plasmids 1 and 3. SNAP-25 expression was induced by IPTG in the case of plasmid 1 but appeared at its maximal level of expression before IPTG addition in the case of plasmid 2. The same pattern of expression of *CeCav*, Munc-18a, Syntaxin-1A, and SNAP-25 was observed when we combined plasmids C with 1, 2, or 3, particularly a clear induction of *CeCav* by AHT and Syntaxin-1A by IPTG in all cases (Fig. S1*C*). The expression of VAMP-2 appeared however not inducible by IPTG in the later cases as compared to the situation without plasmid C (compare Fig. S1*C* with Fig. S1*B*). Given that membrane fusion was previously shown to depend strictly on the presence of Syntaxin-1A and that the expression of this protein was clearly induced by IPTG in spite of some leakage in T7 polymerase synthesis, we did not attempt to modify our pRSFDuet-1–derived vectors in order to get a tighter control of Munc-18a, SNAP-25, and VAMP-2 expression.

**Figure 2:**
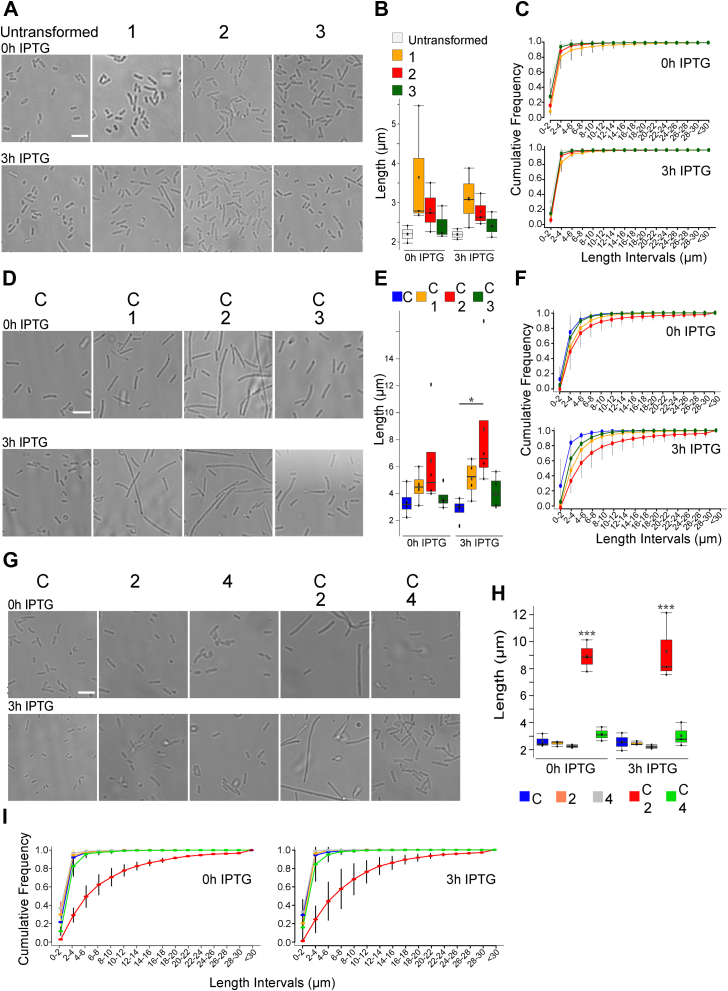
**Increased size of bacteria coexpressing Caveolin and synaptic SNAREs.** The morphology of bacteria transformed with the indicated plasmids was analyzed both at times 0 and 3 h of IPTG exposure, in the absence (*A*–*C*) and in the presence of caveolin synthesis (*D*–*F*), after overnight culture in the presence of AHT. SNARE expression induced bacterial cell elongation only in the presence of *CeCav* expression. *A*, *D*, and *G*, phase contrast microscopy appearance of bacteria (*A*, *D*, and *G*). Note the abundance of the elongated morphology acquired by cells coexpressing caveolin and SNAREs in the absence of Munc-18a. Boxplot of the quantification of cell length for all combinations of expressed proteins (*B*, *E*, and *H*) and as cumulative frequency distribution means ± SD (*C*, *F*, and *I*). The effect of TeNT-LC expression in the absence of Munc-18a synthesis (*G*–*I*) was analyzed in bacteria containing caveolin or not. Note that cell elongation is prevented upon TeNT-LC expression in the presence of caveolin (compare *C*, *F*, and *I*). Bacteria length was measured with ImageJ software using the plug-in ObjectJ. Data were obtained from four (*E* and *H*) and three (*B*) independent experiments, and between 500 and 1000 cells were measured for each condition. Bar represents 5 μm. *B* and *H*, one-way ANOVA with Tukey’s multiple comparison test. *E*, ANOVA orthogonal contrast. (∗*p* ≤ 0.05; ∗∗∗*p* ≤ 0.001). Dots in boxplot represent the mean value of each experimental replicate and *black squares* correspond to the mean of the experimental replicates. AHT, anhydrotetracycline; SNARE, soluble N-Ethylmaleimide–sensitive attachment protein receptor; TeNT-LC, light chain of tetanus neurotoxin.

Using the same experimental setup, we then characterized the protein complexes formed by Syntaxin-1A by a pull-down assay *via* its 6xHis tag using Ni-NTA–coupled beads. In the absence of *CeCav*, we found that Syntaxin-1A could capture Munc18-a, SNAP-25, and VAMP-2 whenever these proteins were expressed. The presence of Munc-18a decreased the amount of VAMP-2 bound to Syntaxin-1A when SNAP-25 was also expressed. Interestingly, the expression of Munc-18a appeared to increase the Syntaxin-1A expression level and the relative efficiency of recovery on the beads (Fig. 1*B*). This latter observation could be related to the fact that the 6xHis tag was added at the N-terminus of Syntaxin-1A, suggesting that Munc-18a binding could help unmasking the tag favoring binding to the beads. Coexpression of *CeCav* by plasmid C did not modify the patterns of expression and the recovery on Ni-NTA beads of Munc-18a, Syntaxin-1A, SNAP-25, and VAMP-2 (Fig. 1*C*). We then assayed for binding of SNAREs and Munc-18a to *CeCav* by isolating the later using its maltose-binding protein (MBP) tag and amylose-coupled beads. We found that *CeCav* could efficiently bind Syntaxin-1A and SNAP-25 but not VAMP-2 (Fig. 2*C*) in good agreement with previous findings (31, 32).

In this cellular system, the formation of putative and distinct complexes cannot be easily characterized, at variance with *in vitro* systems, due to the lack of control on the respective concentrations of exogenously expressed proteins. We considered that the most important issue was to ascertain the presence in induced cells of the canonical synaptic SNARE complex built up with Syntaxin-1A, SNAP-25, and VAMP-2. In a different approach, we took advantage of the presence of His6 and StrepTags present respectively on Syntaxin-1A and VAMP-2 to perform sequential affinity chromatography. In this case, material associated to His6-syntaxin, first bound to Ni-NTA matrix, then specifically eluted and submitted to a chromatography on StrepTactin-coupled beads to capture Syntaxin-1A-VAMP-2 prior to specific elution by desthiobiotin (Fig. S2). The presence of the three proteins in the desthiobiotin eluate demonstrates that in cells containing plasmids C and 2 and after AHT and IPTG induction, Syntaxin-1A, SNAP-25, and VAMP-2 are engaged in a tripartite complex. Together, these results demonstrate that it is possible to reconstitute the expected synaptic complex in the cellular context of *E. coli*. and to obtain inducible expression of *CeCav* and Syntaxin-1A and to reconstitute in the same context of previously demonstrated protein complexes formed by these proteins.

By examining the morphology of the cells at defined steps of the imposed induction protocol, we noticed that bacteria had distinct sizes depending on the combination of plasmids. Because of these changes in phenotype, we asked if expression of *CeCav* with all synaptic SNAREs and Munc-18a or a subset of these proteins would induce a morphological change in *E. coli* resulting from a fusion of the cisternae with the plasma membrane driven by the SNAREs. We first transformed the cells with plasmids 1, 2, or 3 only and found no effect on cell shape and length before and after addition of IPTG (Fig. 2, *A*–*C*). When we first induced expression of *CeCav* by AHT and then added IPTG to cells transformed with plasmid 2, we observed close to a tripling of average cell length, compared to cells transformed with plasmid C alone (Fig. 2, *D*–*F*). This effect was much less pronounced when Munc-18a was also expressed using plasmid 1 or SNAP-25 was omitted using plasmid 3 (Fig. 2, *D*–*F*). We expressed *CeCav* alone (plasmid C), the synaptic SNAREs alone (plasmid 2), and *CeCav* together with synaptic SNAREs and TeNT-LC (plasmid 4) and compared these controls to the coexpression of *CeCav* together with synaptic SNAREs (plasmids C + 2). Only the latter condition was found to induce a robust increase of cell length (Fig. 2, *G*–*I*). Importantly, we found that there was no correlation between the average length values and the final absorbance of the culture at time 3 h of IPTG exposure. We also observed that the growth rates for the distinct step of culture could not be strictly controlled (Fig. S3, *A* and *B*). Several experiments confirmed that absorbance of bacterial cultures is not correlated to the cell phenotype. Only the combination of the plasmids used was able to impose the cellular phenotype reproducibly as quantified in Figure 2. Therefore, we systematically checked the expression of the tested proteins and found similar patterns as shown in Figure 2 (Fig. S4, *A* and *B*). In addition, we checked the expression of TeNT-LC and efficiency of cleavage of VAMP-2 (Fig. S4*C*). The TeNT-LC was detectable even without IPTG induction and accordingly, VAMP-2 expression was obliterated in the presence of the toxin (plasmid 4, Fig. S4*C*). These experiments show that expression of *CeCav* and synaptic SNAREs generated an increased length phenotype. The lack of effect in the absence of SNAP-25 and in the presence of TeNT-LC, *i.e.*, in the absence of VAMP-2, nicely demonstrated that the elongated phenotype correlated with the expression of a fully functional synaptic SNARE complex. Interestingly, Munc-18a behaved here as an inhibitor because the increased cellular length phenotype was absent when Munc-18a was expressed.

**Figure 3:**
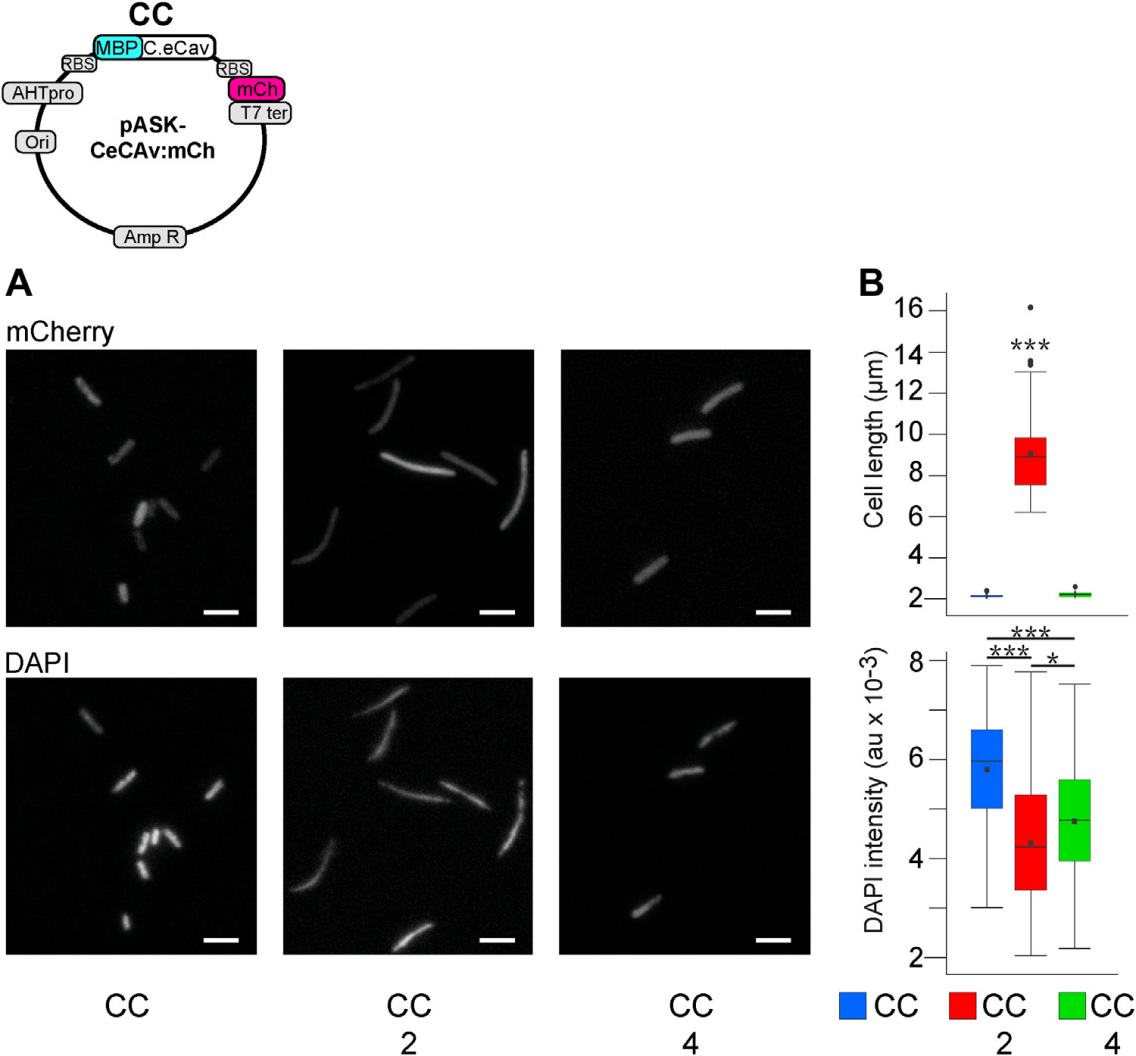
**Invariance of DNA cellular content in elongated bacteria.** Map of bicistronic plasmid CC (pASK background) driving expression of *CeCav* and mCherry. Bacteria transformed with the indicated plasmids combinations were analyzed at time 3 h of IPTG exposure in the presence of caveolin synthesis after overnight culture in the presence of AHT as in Figure 2. *A*, representative fluorescence images of bacteria after fixation, illustrating either cell morphology as seen in epifluorescence (*upper row*, mCherry distribution, compare with Fig. 2*G*) or DNA content after DAPI staining and recording of raw z-stack images in confocal microscopy (*lower row*, DAPI,). *B*, boxplots of the quantification of cell length, for the corresponding combination of expressed protein (*upper panel*) using mCherry images. Note a quasi-identity with Figure 2*H*. Boxplot of total cellular DAPI fluorescence (*lower panel*) in arbitrary units. *Black squares* in boxplot represent the mean value. Data were obtained from three independent experiments, and between 100 and 150 cells were measured for each condition (∗*p* ≤ 0.05; ∗∗∗*p* ≤ 0.001). Bar represents 5 μm. AHT, anhydrotetracycline.

Increased cell length in *E. coli* has previously been associated with cell division failure such as that seen in divisome mutants (33). Here, it thus appeared critical to assay whether or not cells expressing synaptic SNAREs and *CeCav* might be defective in cell division. To this end, we used DAPI staining to assay DNA content and replication. We further constructed a bicistronic pASK plasmid allowing the coexpression of mCherry together with *CeCav* in order to measure both the length of the cells and their DNA content by fluorescence microscopy. These experiments confirmed the increased length in *E. coli* coexpressing mCherry, *CeCav*, and synaptic SNAREs but not mCherry, *CeCav* only, and not mCherry+*CeCav*+synaptic SNAREs and TeNT-LC (Fig. 3, *A* and *B*, top panels). In the same cells, in the expression of *CeCav*, mCherry, and synaptic SNAREs, with or without TeNT-LC, DAPI intensity was slightly albeit significantly decreased compared to CeCav+mCherry only (Fig. 3, *A* and *B*, bottom panels). Altogether, these experiments indicated that the increased cell length observed in *E. coli* expressing *CeCav* and synaptic SNAREs did not coincide with DNA replication and thus does not result from impaired cell division.

**Figure 4:**
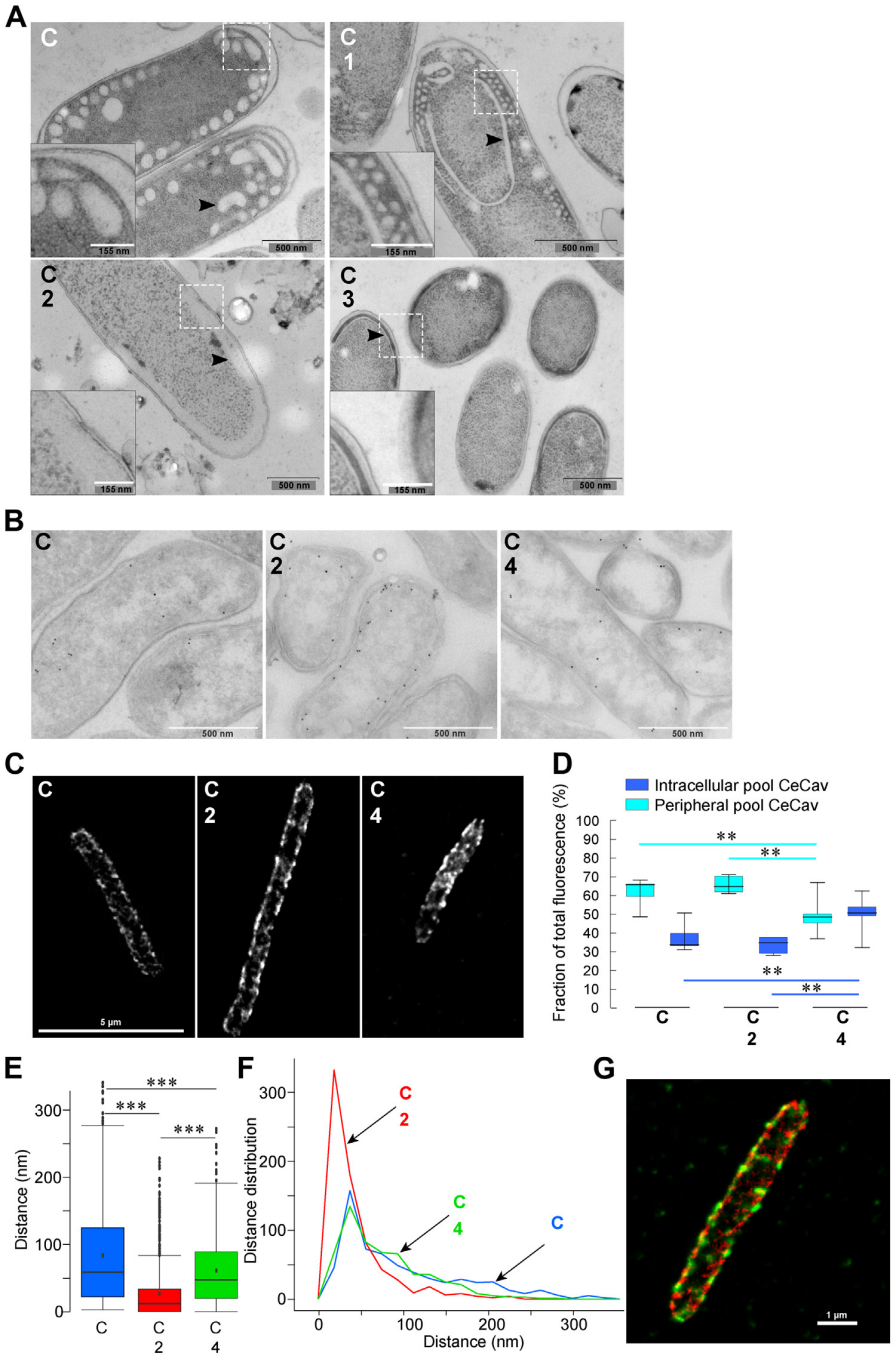
**Decreased cisternae upon synaptic SNARE induction.** Cells were analyzed after overnight production of caveolin (AHT induction) and a 3-h SNARE synthesis (IPTG induction) using the indicated plasmids. *A*, representative EM images of high-pressure fast-frozen cells. *Arrowhead* points cytoplasmic cisternea. Inset (*black border square*) represent the *dotted square* area containing cisternae-rich regions in the cell. Bar represents 500 nm, zoom magnification bar represents 155 nm. *B*, chemically fixed, cryosectioned cells were examined after protein expression from indicated plasmids. Immunogold labeling of whole cells with anti-MBP antibody was performed. Note the preferential localization of the gold particles close to the plasma membrane when caveolin and SNAREs are expressed in the absence of TeNT. Careful observation of the cell periphery after the synthesis of *CeCav* by EM did not reveal contacts of the cisternae and the PM with visible pores allowing continuity between periplasm and cisternae lumen. Bar represents 500 nm. *C*, STED Imaging of *CeCav* (anti-MBP antibody) expressed in the same conditions than (*B*). *D*, distribution of *CeCav* in the peripheral and intracellular pools as defined in Experimental procedures and normalized to the total fluorescent intensity of proteins. *E* and *F*, distribution of gold particles in *Escherichia coli* cells immunolabeled with anti-MBP antibody and super-resolution microscopy of *CeCav* and Syntaxin-1A. The distance between particles and the plasma membrane was measured in sections as illustrated in (*B*), for the indicated plasmid combinations. Boxplot of the quantification of the distance between particles and the plasma membrane (*E*) and as frequency distribution means ± SD (*F*). Data were obtained from three independent experiments Note the decrease of the gold particle to plasma membrane distance only when caveolin and SNAREs are expressed in the absence of TeNT (combination C + 2). *G*, STED imaging of Star *Green*– and Star *Orange*–labeled *CeCav* and Syntaxin-1A, respectively, in cells transformed with plasmids C + 2 shown in (*C*), central panel. Note the partial colocalization of the two proteins in clusters mainly present at the cell periphery. Bar represents 1 μm. (One-way ANOVA test, ∗∗*p* ≤ 0.01; ∗∗∗*p* ≤ 0.001). AHT, anhydrotetracycline; MBP, maltose-binding protein; SNARE, soluble N-Ethylmaleimide–sensitive attachment protein receptor.

We then wondered if the elongated cell phenotype induced by coexpression of *CeCav* and synaptic SNAREs would be associated with the loss of cisternae. Indeed, fusion of *CeCav* cisternae by the action of SNAREs would be expected to induce homotypic fusion of the cisternae and fusion with the plasma membrane. This would result in disappearance of the cisternae from the bacteria cytoplasm upon expression of synaptic SNAREs. To test this hypothesis, we used electron and super-resolution microscopies. After the synthesis of *CeCav*, EM revealed the presence of clear cisternae with a membrane not appearing to be in continuity with the plasma membrane, upon careful observation of the cell periphery (Fig. 4*A*, plasmid C). EM observation showed that only the expression of Syntaxin-1A, SNAP-25, and VAMP-2 using plasmid 2, together with that of *CeCav*, resulted in massive decrease of the presence of cisternae in the cytoplasm (Fig. 4*A*). Cisternae were still observed in the presence of *CeCav* only (plasmid C) and *CeCav* plus synaptic SNAREs and Munc-18a (plasmids C + 1, Fig. 4*A*). Interestingly, when SNAP-25 was omitted (plasmids C + 3), the cisternae appeared apposed to the plasma membrane (Fig. 4*A*), suggesting a docking without fusion phenotype. To further understand the fate of caveolin cisternae, we labeled the protein and carried out immuno-EM observations. Gold particles were found to distribute through the cytoplasm in cells expressing *CeCav* alone (plasmid C) and in very close proximity with the plasma membrane when synaptic SNAREs were coexpressed (plasmids C + 2, Fig. 4*B*). As a control, coexpression of TeNT-LC (plasmid 4) prevented the effect of synaptic SNAREs (Fig. 4*B*). We quantified the distance of the immunogold particles to the plasma membrane and found that expression of synaptic SNAREs decreased the average distance 3-fold (Fig. 4, *E* and *F*). Because immunogold labeling could be biased by detecting only a subset of Cav, we also used classical immunostaining and super-resolution STED microscopy. We found that the MBP staining corresponding to caveolin identified a pool at the most peripheral limit of the cell and a more intracellular pool when *CeCav* was expressed alone (plasmid C, Fig. 4, *C* and *D*). The intracellular pool of caveolin was decreased upon coexpression of synaptic SNAREs (plasmids C + 2, Fig. 4, *C* and *D*) and this effect was reversed by TeNT-LC expression (plasmids C+4, Fig. 4, *C* and *D*). In agreement with our finding that Syntaxin-1A and *CeCav* interact (Fig. 1*D*), we found that Syntaxin-1A and *CeCav* immunolabelings were punctuate and that some puncta of Syntaxin-1A and *CeCav* showed clear colocalization (Fig. 4*G*). In essence, both our EM and super-resolution microscopy observations are compatible with the hypothesis that coexpressing functional synaptic SNAREs together with *CeCav* leads to the consumption of caveolin-induced cisternae, unconnected to the plasma membrane, by their homotypic fusion and/or fusion with the plasma membrane.

## Discussion

In this article, we have described a new synthetic biological approach to reconstitute the function of synaptic SNAREs in *E. coli*. We took advantage of the cisternae-forming effect of *CeCav*1 expression in *E. coli* to search for the effect of coexpressing synaptic SNAREs, which constitute the prototypical nanomachinery of membrane fusion and found that (1) synaptic SNAREs formed bona fide tripartite complexes when they were coexpressed in the absence or in the presence of Cav, (2) synaptic SNAREs expression enhanced bacterial length only when the three synaptic SNAREs were coexpressed in the presence of Cav and not in the presence of TeNT-LC, (3) expression of synaptic SNAREs did not lead to cell division arrest or DNA replication alteration, and (4) conditions which led to enhanced length correlated with the consumption of cytoplasmic cisternae as seen both by EM and super-resolution microscopy.

**Figure 5:**
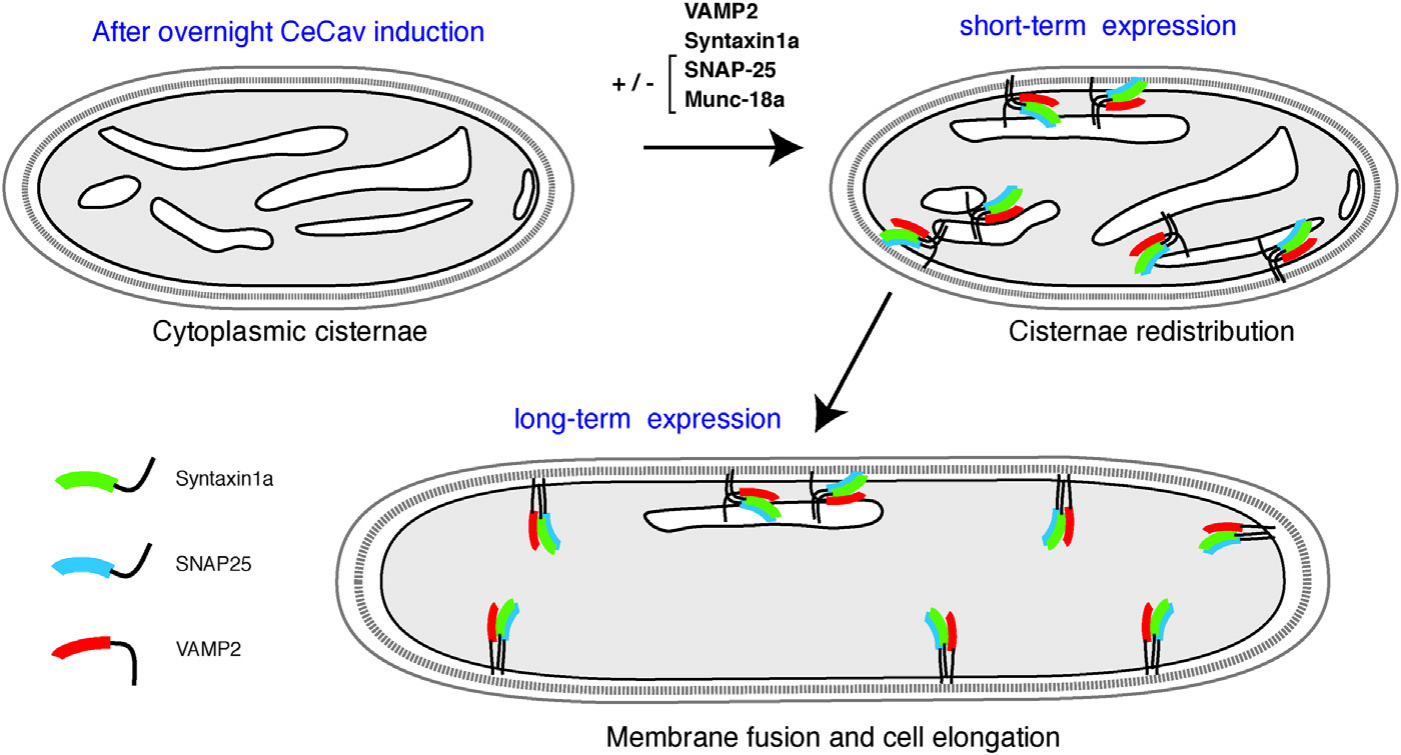
**A model of SNARE-mediated intracellular membrane fusion leading to elongation in Caveolin-expressing *Escherichia coli*.** Bacteria are cotransformed with pASK-CeCav and one of the pDuet-derived plasmids shown in Figure 1*A*. The production of intracytoplasmic membrane invaginations (cisternae) originating from the plasma membrane (*dark line*) is first obtained after the selective expression *CeCav* (AHT induction). In a second phase, v- and t-SNAREs are induced by IPTG and they combine into SNARE complexes. Any functional trans-SNARE complex would mediate fusion of cisternae with cisternae and of cisternae with the plasma membrane. This intracellular membrane mobilization would result in plasma membrane expansion and elongated bacteria. AHT, anhydrotetracycline; SNARE, soluble N-Ethylmaleimide–sensitive attachment protein receptor.

The fusogenic role of synaptic SNAREs has been demonstrated by a large set of experiments using *in vitro* fusion of artificial membranes. From these experiments, it is known that the presence of both VAMP-2, Syntaxin-1A, and SNAP-25 is required (34). Here, we found that *E. coli* cellular elongation and disappearance of cytoplasmic caveolin-cisternae were observed only when we coexpressed VAMP-2, Syntaxin-1A, and SNAP-25 and not when SNAP-25 was omitted. Our biochemical data further showed that Syntaxin-1A interacted with SNAP-25 and VAMP-2 when the three synaptic SNAREs were coexpressed, thus suggesting that the observed tripartite SNARE complex was functional. It is also established that cleavage of SNAREs by CNTs blocks membrane fusion of artificial membranes (35). Here, we found that *E. coli* elongation was not observed and cytoplasmic cisternae were maintained when TeNT-LC was coexpressed with synaptic SNAREs. The role of Munc-18a has been the matter of debate with both evidence for negative and positive roles in membrane fusion. The positive role of Munc-18a has been shown in the context of preassembly of the synaptic SNARE complex (34, 36). The inhibitory role of Munc-18a was however shown when Munc-18a was preincubated with Syntaxin-1A–bearing liposomes and the fusion assay with VAMP-2-liposomes and SNAP-25 was started without room temperature preincubation (34). Here, the *E. coli* elongation effect of synaptic SNAREs was not observed when Munc-18a was coexpressed. Interestingly, in our system, Munc-18a was expressed before the addition of IPTG, whereas the expression of Syntaxin-1A was more tightly regulated and clearly enhanced upon induction (Fig. S1, *B* and *C*). Thus, it is reasonable to think that newly synthesized Syntaxin-1A molecules were trapped in complex with preexisting Munc-18a leading to an inhibited Syntaxin-1A in our setup. In support of this hypothesis, we found less VAMP-2 coprecipitated with Syntaxin-1A in the presence of Munc-18a. One possible caveat of our assay was that SNAREs would form cis–SNARE complexes as soon as they would be synthesized. We think that this was not the case because of the effect of Munc-18a as discussed above and the fact that TeNT-LC was able to cleave VAMP-2 which is only possible when VAMP-2 is not associated with Syntaxin-1A and SNAP-25 (37). In addition, the capacity of Cav to associate with Syntaxin-1A and SNAP-25 but not VAMP-2, as confirmed here, may allow for the formation of Cav/Syntaxin-1A/SNAP-25 complexes and of a typical formation of target-SNARE composed of Syntaxin-1A and SNAP-25. The latter would then be the target of VAMP-2-carrying membranes and formation of the typical synaptic SNARE complex would lead to intracellular membrane fusion (Fig. 5). Formation of these synaptic SNARE complexes would induce fusion of both cisternae with cisternae (homotypic fusion) as well as heterotypic fusion of cisternae with the plasma membrane. This would lead to an increase in cell surface and elongation as shown here (Fig. 5). We do not think that expression of synaptic SNAREs acted on the cell division mechanism of *E. coli* because we found no accumulation of DNA in any of the conditions tested. Indeed, it is well known that Min and FtsZ proteins control abscission and mutating FtsZ also generated elongated ‘eel-like’ bacteria (38, 39). In addition, such a mechanism would not explain our results because the elongation was observed only when synaptic SNAREs were coexpressed with *CeCav*, not observed when we added Munc-18a or TeNT-LC, and was not associated to cell viability defect. Furthermore, signaling cascade from DNA replication to cell division, so-called ‘‘adder’’ behavior, was likely not engaged in our conditions because the cells coexpressing *CeCav* and synaptic SNAREs grew more than the +30% of cell volume triggering this behavior (40). Altogether, this suggests that consumption of cisternae was likely the sole event leading to increased cell length upon coexpression of *CeCav* and synaptic SNAREs.

In order to make cisternae upon expression of *CeCav*, bacteria need to upregulate lipid synthesis. Interestingly, both in the original study (26) and here, the expression of only *CeCav* did not lead to detectable changes in cell size. The most likely explanation is related to the intrinsic properties of Cav to bend membranes and generate cisternae and vesicles in an ‘endocytic-like’ activity compensated by the synthesis of new lipids to prevent cell shrinkage (26). The fact that expression of synaptic SNAREs, with a full induction a few hours after *CeCav*, was able to consume the cisternae suggests that the fusogenic activity produced by SNAREs engaged a larger membrane area than the endocytic-like activity of Cav, *i.e.* that SNARE activity dominates over that of caveolin when all proteins are expressed. Our data further suggest that the cisternae membrane area generated by *CeCav* expression likely corresponded to ∼2 to 3 times the plasma membrane because this corresponds to the extension rate we observed upon of the synthesis of synaptic SNAREs after that of *CeCav*. Experiments using different conditions and times will allow to further dissect this mechanism.

In conclusion, we propose here a new assay to study the function of SNAREs. This assay is based on the expression of full-length SNAREs, their partners, and regulators, does not need protein purification, and allows for an easy readout of membrane fusion by measurement of bacterial cell length. Beside the reconstitution of the fusogenic synaptic SNARE complex in the bacteria, by essence the system proposed consists in imposing the formation of a nonnatural compartment acting as membrane donor in a fully controllable exocytic event. In the context of membrane fusion, it can be used to study not only events involving other SNARE associations but different fusion machineries. We think that our synthetic biological approach opens new avenues for structure-function studies of SNAREs in a cellular environment and the search of drugs directly acting on SNARE-mediated membrane fusion, possibly in combinations with emerging membrane reconstitution technologies. It further supports the hypothesis that a small subset of genes might have been sufficient to allow for the appearance and functionality of internal membranes during evolution from prokaryotes to eukaryotes since cisterna formation and its fusion activity could be reconstituted here in bacteria by expressing only four proteins. Further studies aiming at reconstituting membrane recycling would allow to move a step further towards reconstituting essential eukaryote properties in prokaryotes.

## Experimental procedures

### Experimental design/study design

The biological system described in this report aims at reconstituting the molecular machinery acting in intracellular membrane fusion encountered in eukaryotic cells. The model proteins used are the synaptic SNAREs, which constitute the synaptic vesicle fusion nanomachinery, with remarkable conservation with other SNAREs operating in membrane fusion from yeast to mammals.

The current system corresponds to a bacterial cell, *E. coli* (BL21(DE3)pLysS), in which several proteins of exogenous origin can be simultaneously produced. The system allows to control on the one hand the synthesis of mammalian synaptic SNARE proteins and on the other hand the biogenesis of intracellular cisternae following the synthesis of *CeCav*. Production of these proteins results from distinct transcription/translation units present in two coexisting plasmids after bacterial transformation. The involved plasmids have therefore different and compatible replication origins and provide different antibiotic resistances allowing double transformant selection.

SNARE proteins are expressed from plasmid pRSFDuet-1; replication origin RSF 1030, kanamycin resistance (aminoglycoside phosphotransferase, KanR). The plasmid pASK-IBA3plus is used for producing *CeCav*; replication origin ColE1/pMB1/pBR322/pUC, ampicillin resistance (β-lactamase, AmpR). The advantage of this combination lies in the separated control of transcription from the two vectors harboring distinct promotors (see below). The double transformant constitute a flexible system: they offer the possibility to express in a nonsimultaneous manner the proteins encoded by the respective vectors and to choose the expression sequence as well as time-delayed expression.

A standard use of transformed bacteria consists in generating cisternae from plasma membrane invagination (internal membrane). As previously described (26), this steps exploits the ability of *CeCav* of forming pleiomorphic intracytoplasmic membrane inclusions. These structures are central to the reconstitution system. They are a reservoir of membrane material which is mobilized in order to increase the plasma membrane area when the synthesis of proteins able to associate in a fusogenic complex is induced. In transformed bacteria, the biological assay includes two steps: the occurrence of intracytoplasmic cisternae is first induced by *CeCav* synthesis. In a second step initiated by inducing synaptic SNARE proteins in a potentially fusogenic combination, one sees a remarkable increase in cell length, coupled with the fusion of intracellular membranes with the plasma membrane.

### Plasmid design, assembly, and verification

All plasmid constructs were assembled using a combination of assembly PCR and traditional restriction enzyme digestion followed by ligation or by Gibson assembly. Restriction enzymes and T4 DNA Ligase were acquired from New England BioLabs and used according to manufacturer’s recommendation. PCRs were performed using either Pfu DNA polymerase supplied by Promega or the Phusion High-Fidelity DNA Polymerase by New England BioLabs, following their recommended protocol. Primers were designed manually and ordered from SIGMA/Invitrogen/Eurofins. When necessary, constructs were edited following QuikChange II Site-Directed Mutagenesis Manual directions. DNA, agarose gel, and plasmid purification steps were performed using the NucleoSpin Gel and PCR Cleanup Kit, the NucleoSpin Plasmid Kit, respectively, all obtained from Macherey Nagel. Visualization of agarose gels was performed using the SYBR Safe DNA Gel Stain, from Invitrogen and visualized in a transilluminator under UV excitation. All inserts were fully verified using GATC Sanger sequencing services.

#### Caveolin–pASK-CeCav expression vector

The cav-1 cDNA from *C. elegans* (*CeCav*) cloned in pNMTMA-CeCav vector has been described (Walser *et al*., 2012). It is a chimeric cDNA coding for a fusion protein MBP-*CeCav* and bearing a His6 tag in the c-terminal position. In the absence of signal peptide, MBP imposes a cytoplasmic accumulation of the chimera.

In the present system, the host vector chosen for the expression of *CeCav* is pASK-IBA3plus (IBA GmbH, Göttingen). This plasmid carries the inducible tetracycline promoter/operator for the regulated expression of the protein (it constitutively produces the TetR transcriptional repressor whose binding on the TetO operator is prevented by tetracycline or doxycycline). Transcription can therefore be induced by AHT that exhibits no antibiotic activity under recommended concentrations used.

After PCR amplification from pNMTMA-CeCav, a MBP-*CeCav* fragment devoid of His_6_ tag was obtained, which included a Stop codon (to avoid StrepTag fusion protein present in the host vector). This fragment was subsequently cloned into pASK-IBA3plus vector using the restriction sites EcoRI and XhoI by conventional cloning techniques.

#### Caveolin + mCherry–pASK-CeCav:mch bicistronic expression vector

A PCR fragment, SDmChe, was first synthesized in which the mCherry ORF was fused downstream to a Shine-Dalgarno sequence. The SDmChe DNA was then inserted in the pASK-CeCav plasmid by classical Gibson assembly method, upstream to the T7 terminator and between the XhoI and HindIII restriction sites, giving a bicistronic pASK-CeCav:mch plasmid. This allowed the simultaneous production of *CeCav* and mCherry upon AHT induction.

#### Polycistronic vectors

The simultaneous cDNAs expression of SNARE proteins and of the associated Munc-18a protein exploits the transcription by the T7 polymerase which allows the synthesis of transcripts of at least 10 kbases. The vector chosen for this purpose is the tetracistronic plasmid pST44 (41) containing a set of four translation cassettes, flanked by a T7 promoter and a T7 termination site. It corresponds to an improved polycistronic expression vector obtained by asymmetric subcloning of translation units (cassettes) (41). The transcription can therefore be induced by IPTG or β-D-galactopyranosyl (1 → 4) D-glucopyranose. The unique transcript produced contains four autonomous reading frames for their translation.

Each cassette thus comprises a translational enhancer (TTAACTTTA) and a Shine-Dalgarno (AAGGAG) sequence for binding the ribosome as well as initiation (ATG) and translation termination (Stop: TAA or TGA) codons. Respectively, immediately upstream or downstream of the ATG or Stop codons is one of the six possible "reporter" (tag) sequences, encoding antigenic sequences, or affinity ligands. In the N-terminal position of the proteins of interest, these labels can be cleavable (TEV protease).

The construction of the various cassettes with the insert of interest is first carried out in vectors of the pST50Trc series by conventional cloning techniques with only the BamHI and NgoMIV restriction sites. This cloning places the cDNA of interest in the same reading phase as the label and the ATG and Stop codons. The recombinant vectors pST50 are both expression plasmids (IPTG induction) and transfer plasmids. They allow the excision of the cassettes, respectively, with the aid of four pairs of restriction sites, XbaI/BglII, EcoRI/HindIII, SacI/KpnI, BspEI/MluI.

The selection of the pST50 vectors as well as the position and the order in which the cassettes are inserted in the pST44 are necessarily coupled. They are imposed both by the restriction map of the five cDNAs involved and by the nature and role of the added "reporter" sequences.

#### Vectors construction of the pST50 series

The SNARE cDNAs: Stx1a (*Rattus norvegicus*), VAMP2 (*Bos taurus*), Snap25 (*R. norvegicus*), and Munc18-1 (Apba1, *R. norvegicus*) were amplified by PCR from plasmids of the laboratory, the fragments included BamHI and NgoMIV restriction sites (or compatible sites when necessary). They were cloned into a pST50 vector by DHFR substitution as follows: Stx1a in pST50Trc1-HISNDHFR, VAMP2 in pST50Trc2-STRNDHFR, Snap25 in pST50Trc3-CBPNDHFR, Munc18-1 in pST50Trc2-DHFRFLAG. At this stage, the sequence of the new constructions was checked. Thus, labeled versions are obtained allowing either affinity isolation: His6-Syntaxin-1A, StrepTag-VAMP-2, CBP-SNAP-25 (cleavable labels) or immunoprecipitation: Munc-18a-FLAG (noncleavable) present in five separate cassettes. The expression of each vector was confirmed after transformation in BL21 (DE3) pLysS bacteria and IPTG (1 mM) induction, followed by electrophoresis and Western Blot immunodetection by specific antibodies.

#### Construction of the pST44 vectors

These cassettes are used to construct the basic pST44 tetracistronic vector. Thus, in pST44, StrepTag-VAMP2 is cloned in position 2, then sequentially CBP-Snap25 in position 3, Munc18-1 in position 4. The cloning of His6-Stx1a in position 1 requires a silent mutagenesis of Snap25 eliminating a site XbaI restriction. The basic vector obtained is then pST44-St.VA.SN.Mu whose sequence was verified. At this stage of construction, the coexpression of the four proteins by the vectors in BL21 (DE3) pLysS bacteria was verified as before.

#### Construction of pRSFDuet-1-St.VA.SN.Mu vector

The plasmid pRSFDuet-1 was developed by Novagen and is part of the laboratory's collection. In our system, obtaining recombinant vectors is facilitated by the presence of the same T7 promoter (promT7) and T7 termination sites (termT7) in pST44 and pRSFDuet-1plasmids. Therefore, the complete transcription unit *promT7-StVASNMu-termT7* belonging to the recombinant plasmids pST44 was transferred into pRSFDuet-1 by Gibson assembly ("Gibson assembly"). The primers used to amplify these fragments were: Forward Cistron (Fwd2),5′- GACTCCTGCATTAGGAAATTAATACGACTCACTATAGGGAGAC) and Reverse Cistron (Rev2), 5′- TCAAATGCCTGAGGTTTCAGCAAAAAACCCCTCAAGACCCG. The plasmid pRSFDuet-1 was opened by PCR using primers Forward pRSF (Fwd), 5′- CTGAAACCTCAGGCATTTGAG and Reverse pRSF (Rev), 5′-ATTTCCTAATGCAGGAGTCGC). The Gibson reaction was performed according to the standard protocol (New England Biolabs). The coexpression of the four proteins from pRSFDuet-1-St.VA.SN.Mu vector was confirmed as before and took place with relative levels of synthesis identical to that of vector pST44.

#### Construction of pRSFDuet-1-St.Va.SN.∗, pRSFDuet-1-St.Va.∗.Mu, and pRSFDuet-1-St.Va.SN.TeNT vectors

Subsequent deletions and/or mutations were performed on the tetracistronic pRSFDuet-1-St.VA.SN.Mu vector in order to express the different combinations of SNAREs proteins (St, VA, ±SN) and including or not Munc18-1 or TeNT (see Fig. 1), used in the present study. The removal of Snap25 from the pRSFDuet-1-St.VA.SN.Mu to generate pRSFDuet-1-St.Va.∗.Mu was done by Gibson assembly using the following primers: Forward Stx/VA ∗ Mu: (5′-ATAAGGTAGGTGATTATATAATGTACAGGTACCAGCGGATAACAATTT) and Reverse Stx/VA Mu:

(5′-TATAATCACCTACCTTATGAAATTCTTTTTCCATCGTCGCTTAGACATATGTAT). In order to generate pRSFDuet-1-St.VA.SN.∗ (minus Munc18-1) from pRSFDuet-1 St.VA.SN.Mu, restriction of KpnI sites flanking Munc18-1 ORF followed by religation was performed. For the experiments in which the tetanus toxin light chain (TeNT) was used to specifically cleave VAMP2, an intermediate plasmid was generated. This was done by PCR mutagenesis of pRSFDuet-1-St.VA.SN.Mu to simultaneously delete Munc18-1 ORF and add AatI and SpeI restriction sites, using primers: Forward DMunc,

5′-GATATACATATGGGATCTGACGTCGGATCTACTAGTGGCGGCGACTACAAGGACG and

Reverse Dmunc, 5′CGTCCTTGTAGTCGCCGCCACTAGTAGATCCGACGTCAGATCCCATATGTATATC. The VAMP2 cDNA was in parallel subjected to point (silent) mutagenesis in order to eliminate a SpeI site. The Tetanus toxin light chain cDNA was amplified by PCR from plasmid pCMV-TeNT LC, using primers introducing AatI and SpeI sites:

Forward Te AatI Up, 5′-GCGGGACGTCGGAATGCCGATCACCATCAACAACTTC and Reverse Te SpeLo, 5′-CGCGACTAGTTTAAGCGGTACGGTTGTACAGG. This fragment was subcloned in the intermediate plasmid. At this stage, the sequence of the new constructions was checked.

Predicted molecular masses of encoded proteins are as follows: MBP-CeCav, 70.17 kDa; His6-Syntaxin 1A, 35.89 kDa; StrepTag-VAMP-2, 15.65 kDa; CBP-SNAP-25, 28.06kDa; Munc-18a-Flag, 68.58 kDa.

#### Bacterial strains and transformation

For all cloning and plasmid maintenance steps, the *E. coli* DH5α strain was used. All experiments were performed using *E. coli* strain BL21(DE3)pLysS, which carry the pLysS plasmid (containing the p15A origin of replication and conferring chloramphenicol resistance) coding for T7 lysozyme to reduce basal transcription from the T7 promoter. Chemically competent bacteria were routinely produced in the laboratory, and transformations were preformed according to classical protocols.

### Culture conditions and protein production

After transformation of BL21(DE3)pLysS cells with the chosen plasmids, the entire bacteria mixture was expanded overnight at 37 °C in 3-4 ml of LB (Miller) medium supplemented with the required antibiotics (80 μg/ml ampicillin, 40 μg/ml kanamycin, 3.4 μg/ml chloramphenicol). This liquid culture was the source of subcultures aimed at protein production and was maintained under orbital shaking to ensure sufficient aeration. As a basis, protein production was carried out according to a two-step protocol. Unless otherwise indicated, the expanded cell suspension was adequately diluted in fresh alpha-MEM medium with nucleosides, supplemented with antibiotics, and grown until an optical density at 600 nm (A_600_) of 0.6 was reached. Then, induction of *CeCav* synthesis was sustained for 3 h at 25 °C in the presence of 0.2 μg/ml AHT. This was followed by four-fold dilution in the same alpha-MEM medium (devoid of AHT) and growth for 1 h at 37 °C to allow for a second logarithmic growth phase and by exposure to 1 mM IPTG for 30 min at 37 °C.

### Samples for biochemical characterization

Samples for biochemical assays were prepared from six A_600_-equivalent aliquots. Cells were recovered from culture samples (six A_600_-equivalent) by centrifugation (3200*g*, 8 min) and resuspended in buffer A (20 mM Hepes pH 7.7, 400 mM KCl, 15% (w/v) glycerol) containing protease inhibitors without EDTA and incubated on ice with 1 mg/ml lysozyme for 5 min. Cells were solubilized with 0.5% (w/v) CHAPS, 1% (w/v) octyl-β-D-glucopyranoside, 25 μg/ml DNase, 0.6 mM MgCl_2_, and 2 mM β-mercaptoethanol in buffer A for 20 min on ice, before the addition of 2 mM EDTA. Solubilized material was recovered in the supernatant (clear lysate) after centrifugation (12 000*g*, 12 min, 4 °C).

The solubilized membrane proteins and complexes were isolated by affinity chromatography, with either Ni-NTA matrix (Superflow Qiagen) or with Amylose resin (New England Biolabs) by batchwise incubation for 1 h at 4 °C. Beads were washed with buffer B (20 mM Hepes pH 7.7, 150 mM KCl, 10% (w/v) glycerol) containing 0.5% (w/v) CHAPS and 1% (w/v) octyl-β-D-glucopyranoside, 2 mM β-mercaptoethanol, 2 mM EDTA, and protease inhibitors. To analyze bound material, washed beads were mixed with 0.33 volumes of four-fold concentrated Laemmli sample buffer, snap-frozen, and stored at −20 °C until analysis. Affinity chromatography on StrepTactin/sepharose was performed according to the manufacturer’s instructions (IBA, GmbH). This chromatography was applied to proteins eluted from Ni-NTA matrix with 250 mM imidazole as described in legend to Fig. S2. Protein samples were analyzed by Laemmli SDS-PAGE using 12% acrylamide gels (acrylamide/bisacrylamide ratio: 37.5/1) followed by electrotransfer on nitrocellulose membrane (Amersham Protran 0.45 NC) at 10 V/cm at 0 °C, in a Tris-glycine buffer, 20% ethanol. For protein immunochemical detection, the following primary antibodies were used: rabbit pAb anti-Maltose Binding Protein (Invitrogen), rabbit pAb Anti-Synaptobrevin2/VAMP2 (Synaptic System), Mouse mAb anti-Syntaxin 1a (HPC-1) (STX01, AbCam), mouse mAb anti-SNAP-25 clone 71.1 (Synaptic System ref number: 111011), rabbit pAb anti-Munc18.1 (Synaptic System), mouse mAb anti-TeNT (light chain–specific T2/13 (42). Horseradish peroxidase–coupled secondary antibodies were revealed using ECL reagents (Amersham, GE Healthcare), and signals were measured and quantified using the ChemiDoc Imaging System (Bio-Rad).

### Samples for microscopy

Following transformation and the first overnight culture at 37 °C (see above), 0.5 ml of cell suspension in LB was processed as indicated above until 0.6 A600 was reached. Bacteria were then grown in alpha-MEM medium containing AHT and antibiotics overnight at 25 °C for Cav induction. Then, a 4-fold dilution with MEM-alpha was applied to the culture, in order to stop AHT induction and to allow cells to reenter a log phase during a 1-h growth at 37 °C. Bacteria were then treated with 1 mM IPTG to express one of the various SNARE/Munc 18/TeNT combinations available for 3 h at 37 °C. Cells were then fixed as detailed below.

### Fixation method

For light microscopy, 0.1 ml of fixation solution (0.01 ml of 25% (w/v) glutaraldehyde in 2.5 ml of 16% (w/v) paraformaldehyde in 0.2× PBS) was added to 500 μl of bacterial culture. The sample was incubated at room temperature for 15 min, then for 15 min on ice. The cell suspension was centrifuged at 3200*g* for 5 min. The pellet was washed two or three times in 1 ml PBS and resuspended in PBS up to 1-2 A_600_-equivalents/ml. Chemically fixed samples for EM were prepared from a two A_600_-equivalent aliquots. Cells were recovered from culture samples (two A_600_-equivalents) and incubated with a final concentration of 4% (w/v) paraformaldehyde, for 10 min at room temperature. The cell suspension was then centrifuged (4000 rpm, 1 min) and the pellet was resuspended in 4% paraformaldehydefor 1 h at room temperature.

### Electron microscopy

Samples for EM were prepared by high-pressure fast-freezing from a 25 A_600_-equivalent aliquots. Cells were recovered from culture samples (25 A_600_-equivalent) and centrifuged at 3200*g* 4 °C for 10 min. The pellet was kept in 20% (w/v) bovine serum albumin (BSA)-PBS on ice until high-pressure fast-freezing was performed using EM PACT2 HPF (Leica Microsystems GmbH), followed by freeze-substitution in a mix of 1 % osmium in acetone using EM AFS 2 (Leica Microsystems GmbH). Samples were subsequently embedded in epoxy resin. Ultrathin (70 nm) sections (Ultracut UC6, Leica) were collected on formvar/carbon-coated copper grids. Sections were then poststained by aqueous 4% uranyl acetate and lead citrate.

Samples were observed in a Tecnai12 (FEI) transmission electron microscope at 80 kV equipped with a 1K×1K Keen View (OSIS) camera.

For immunolocalization, samples were prepared by the Tokuyasu method. Cells were fixed with 4% paraformaldehyde in PBS, washed with PBS, centrifuged and embedded in gelatin 10%. Gelatin cell pellets were cut in small cubes and cryoprotected by infusion in 2.3 M sucrose at 4 °C overnight. Sample cubes were mounted on specimen pin and plunge-frozen in liquid nitrogen. Ultrathin (70 nm) sections (Ultracut UC7-FC7, Leica Microsystems GmbH) were collected on formvar-coated nickel grids. For immunolabeling, sections were blocked in blocking solutions (Aurion), incubated in rabbit anti-MBP antibody (1/50, Thermo Fischer Scientific), washed in PBS with 1% BSA, and labeled with goat anti-rabbit or goat anti-mousse 10 nm gold conjugate IgG (1/20, Aurion), followed by washes in PBS and water and staining in uranyl acetate 0.4%/methyl cellulose 1.8%.

Samples were observed in a Tecnai12 (FEI) transmission electron microscope at 120 kV with OneView 4K×4K (Gatan) camera.

### Quantification of immunogold-particle distance to the plasma membrane

Immunogold-labeled images (using an MBP antibody) were acquired using a Tecnai12 transmission electron microscope. The distance to the plasma membrane of positive particles in the cytoplasm were measured using ImageJ software, https://imagej.nih.gov/ij/. The frequency distribution of particle distance (in nm) was plotted using R software, https://www.r-project.org/. Between, 35 to 40 cells were analyzed per condition.

### Measuring cell lengths

Images of fixed cells were acquired using a Leica DMLA2 microscope and the length of bacteria were measured using the ObjectJ plugin of ImageJ (Rasband, W.S., ImageJ, http://imagej.nih.gov/ij/; Vischer, N.O.E, ObjectJ, http://simon.bio.uva.nl/objectj). The plugin was set to automatically measure all cells in each field. Each treated photograph was examined visually, and incomplete cells and contaminating materials were manually discarded. Cells that were too long for ObjectJ were manually measured. To create the histogram, data were transferred to Excel program and the frequency distribution was plotted as relative frequency. To ensure statistical significance, more than 1500 cell lengths were used for each condition. For cells carrying the pASK-CeCav:mcherry plasmid (CC), dual–color images were obtained after fixation followed by 1.8 μM DAPI staining. mCherry images were used for delineating cell cytoplasm. Length measurements were carried out on images (650 nm filter) from a Leica DMRA2 microscope (objective 100×) using ImageJ set at major measurement setting. Only cells longer than 2 μm were analyzed.

### Fluorescence microscopy

Coverslips were treated with 1 mg/ml poly-L-lysine in 0.1 M borate buffer pH 9.5 for 5 min at room temperature and washed with distilled sterile water. Fifty microliters of fixed cell suspension were dropped on a cover slip, incubated for 10 min, then washed with PBS. Cells were treated with 2 mg/ml lysozyme in 25 mM Tris–HCl pH 8.0, 50 mM glucose, 10 mM EDTA for 7 min at RT, washed with PBS, and incubated for 2 min with 0.1% (w/v) Triton in PBS (PBS-T). Cells were blocked with 2% (w/v) BSA in PBS-T for 20 min and incubated for 90 min with primary antibody in 2% (w/v) BSA 0.1% Triton PBS. Cells were washed 3 to 4 times with PBS and incubated with secondary antibody in PBS-T containing 2% BSA for 1 h and washed 3 to 4 times with PBS. Coverslips were mounted in 5 μl of ProLong Gold (Thermo Fisher Scientific, catalog number: P10144). Images were acquired using a Leica Leica SP8 – STED 3× microscope. Confocal images of fixed cells stained with DAPI (1.8 ⎧M, 10 min) were acquired (450 nm) using a Leica SP5 STED CW microscope. Ten z-stack images were taken per condition from three experiments (N = 3), and 40 cells were selected from each condition. Only objects clearly identified as cells were analyzed using ImageJ set at Mean. Acquisition of double-staining images (mCherry + DAPI) of *E. coli* cells (Fig. 3) was carried out with the Leica DMRA2 microscope (objective 100×).

### STED imaging

Dual-color imaging of Cav and Syntaxin proteins in fixed cells was conducted on a LEICA TCS SP8 gated STED 3× super-resolution microscope (Leica Microsystems-equipped with a tunable white light laser, a continuous 592 nm, and a pulsed 775 nm depletion lines). Stacks (around 2 μm) of images were acquired using a glycerol-immersion 93× objective (NA 1.3) and gated HyD detectors (set at 100%). Cav was labeled with Star Green (Abberior GmbH), and the 592 nm depletion line was set between 40% to 50% with 20% of the power redirected to the Z donut to generate Z resolution of around 230 nm. In the presence of Syntaxin, we labeled these proteins with Star Orange (Abberior GmbH). To generate STED images, the pulsed 775 nm laser line was used at 60 to 80% with 20% redirected to the Z donut to generate a Z resolution of 100 nm. Gated HyD detectors adjusted between 0.4/0.5 to 6 nanoseconds were used to acquire Cav (excited at 495 nm at 5% maximal laser power) and Syntaxin 1 (excited at 598 nm at 3.5% maximal laser power). While carrying dual-colors STED imaging, pixel size was optimized for Syntaxin channel acquisition (15–20 nm XY).

### Image analysis and data analysis

Raw STED images obtained using LEICA TCS SP8 gated STED 3× were subjected to deconvolution using Huygens (SVI). Parameters for deconvolution were kept similar for every condition. Deconvolution was performed separately for each channel using a maximum of 40 iterations. To validate the deconvolution approach, deconvolved images were compared to raw STED images processed with background subtraction and Gaussian blur filter (sigma value to three pixels). Image processing was done using Fiji (43). Images are shown after brightness and contrast adjustment and application of a 2D Gaussian blur filter (sigma value: 0.2–0.5). Prior to analysis, dual-color images were registered using MultistackReg (v1.45). This plugin is based on Turbo reg plugin (44). To measure the distribution of Cav at the membrane (peripheral pool) and in the cell interior (intracellular pool), we used a combination of morphological operation to extract cell contour and a band of 3μm thick was defined to delimitate the membrane region and the subsequent central region corresponds to the cell interior. Distribution was normalized to the total fluorescent intensity of proteins.

### Statistical analysis

Unless otherwise stated, all statistical analysis was performed with the R software. In Figure 1, data from different replicate experiments was standardized as follows: ‘‘std value (x) = (x – sample mean)/sample standard deviation”. Statistical analysis was performed using one-way ANOVA test (Figs. 1, 3 and 5) followed by a Tukey’s HSD test and *p* values below 0.05 considered significant. In some instances (Fig. 2), one-way ANOVA test per time condition was performed, followed by a Tukey’s HSD test (Fig. 2, *B* and *H*) and ANOVA orthogonal contrast with the control condition “C” (Fig. 2*E*).

The lower and upper hinges of all boxplots correspond to the first and third quartiles, respectively (the 25th and 75th percentiles). The upper whisker extends from the hinge to the largest value no further than 1.5 ∗ IQR from the hinge (where IQR is the inter-quartile range or distance between the first and third quartiles). The lower whisker extends from the hinge to the smallest value at most 1.5 ∗IQR of the hinge. Data beyond the end of the whiskers are called ‘‘outlying’’ points and are plotted individually.

## Data availability

All data, code, and materials used in the analyses is available to any researcher for purposes of reproducing or extending the analyses *via* materials transfer agreements. All data are available in the main text or the supplementary materials.

## Supporting information

This article contains supporting information.

## Conflict of interest

The authors declare that they have no conflicts of interest with the contents of this article.

## Supporting information

Supplemental data

FigureS1

FigureS2

FigureS3

FigureS4
