# Supplementary material for: A synthetic organelle approach to probe SNARE-mediated membrane fusion in a bacterial host
Source: J Biol Chem. 2023 Feb 3;299(3):102974. doi: 10.1016/j.jbc.2023.102974 (PMC10011478; doi:10.1016/j.jbc.2023.102974)
Supplement: Supplemental data [file mmc1.doc]

Title: A synthetic organelle approach to probe SNARE-mediated membrane fusion in a bacterial host.

**Authors**

Soledad Ferreras, Neha Pratap Singh, Remi Leborgne, Philippe Bun, Thomas Binz, Robert Parton, Jean-Marc Verbavatz, Christian Vannier and Thierry Galli

**Supplemental figures Legends**

Fig. S1. **Optimization of protein expression in *E.coli*.** (A) Generation of intracytoplasmic cisternae upon AHT exposure of cells transformed with plasmid C only. High-pressure fast-freezing of pelleted cells was performed following culture at 25 or 37°C for the indicated times. Arrowheads indicate membrane enclosed structure with clear lumen which are not observed in cells not synthesizing *CeCav*. (B, C) Time-course of specific immunoreactivity expression of proteins encoded by separate pDuet vectors (B, plasmids 1, 2, 3) or by their combinations with pASK vector (C, plasmids C+1, C+2, C+3), upon induction with AHT and/or IPTG as described in Materials and Methods section. Molecular weights are indicated (kDa).

Fig. S2. **Sequential capture of SNAREs via His6 and StrepTag affinity chromatography.** Bacteria containing plasmids C and 2 were used (3 A600-equivalents) after AHT and IPTG induction. After cell pelleting and resuspension all steps are conducted at 4-6°C. (A) The cell lysate (600 µl) was loaded twice onto 75 µl of Ni-NTA matrix in a 0.8-ml column for a period not exceeding 30 min. After washing of the beads as described in Experimental procedures, His6-syntaxin-containing complexes were eluted with 0.25M imidazole in the same buffer. After a two-fold dilution without imidazole the eluate (E1) was then loaded twice onto 75 µl of StrepTactin matrix in a 0.8-ml column under the same timing conditions. After washing, StrepTag-VAMP2-containing complexes were eluted (E2) with 10mM desthiobiotin. For SDS-PAGE 1.6% (Lysate, L) and 6 and 12% (Eluates, E1 and E2, respectively) of fractions were analysed after heating at 95°C for 5 min. Indicated SNARE proteins were detected after Western blotting by ECL using anti-Syntaxin-1a, anti-SNAP-25 and anti-VAMP2 antibodies as in Figure 1.

Fig. S3. **Growth of BL21pLysS transformed by the various plasmid combinations upon IPTG induction.** (A) Variation of absorbance A600 during IPTG exposure of four separate experiments for the main plasmid combinations pASK-CeCav + versions 1 to 4 of pRSFDuet-1 and corresponding to the morphological analyses reported in figure 2. The growth rates from time 0 to 3h IPTG for a defined plasmid combination are not conserved over experiments and do not strictly correlate with final phenotypes. (B) Four experiments (a-d) are grouped showing a more homogenous growth of bacteria containing either plasmid 1 or plasmid 2 alone. In one experiment however growth was more rapid for both situations for an unknown reason (Black square and circle).

Fig. S4. **Immunoblots of cell samples from cultures used for cell length determinations**. Samples were obtained from experiments of figure 2. Cell content was analyzed after 0 and 3h exposure to IPTG, following overnight growth in the presence of AHT, in bacteria transformed with the indicated plasmid combination, without (A) or with (B) plasmid C, respectively. Substitution of TeNT for Munc-18a (C) was used to inactivate VAMP2. Note that no VAMP2 immunoreactivity was observed whether caveolin was expressed or not (compare conditions 4 and C4 in (C) with conditions 3 in (A) and C3 in (B). Molecular weights are indicated (kDa).
