# Supplementary figures and images for: A synthetic organelle approach to probe SNARE-mediated membrane fusion in a bacterial host
Source: J Biol Chem. 2023 Feb 3;299(3):102974. doi: 10.1016/j.jbc.2023.102974 (PMC10011478; doi:10.1016/j.jbc.2023.102974)

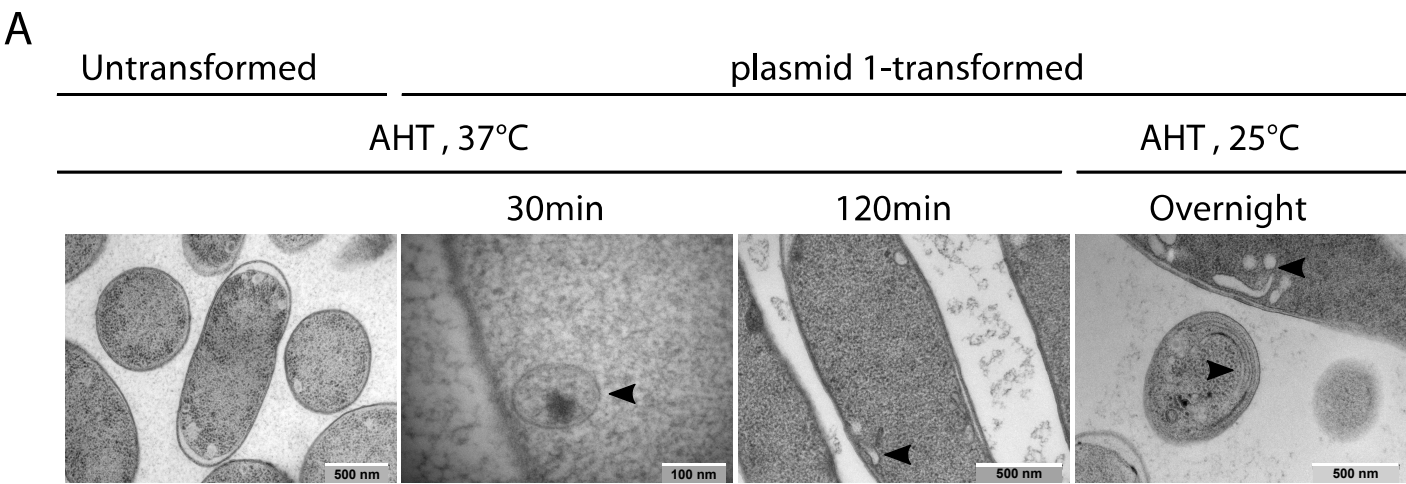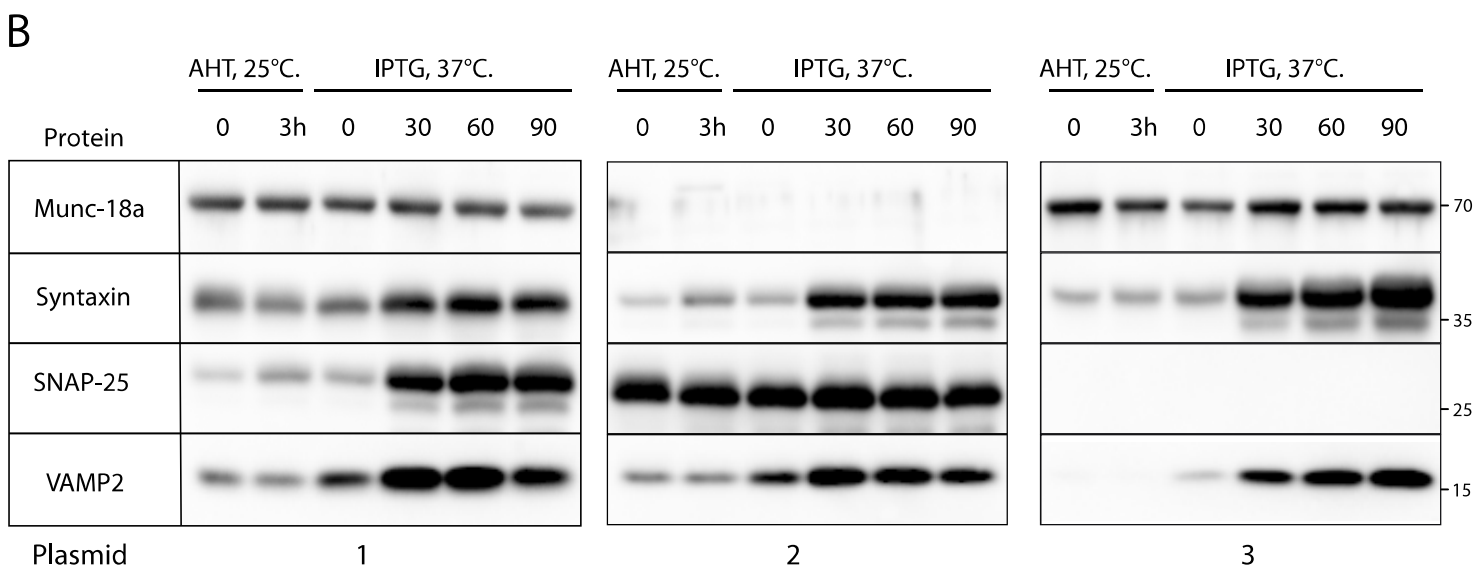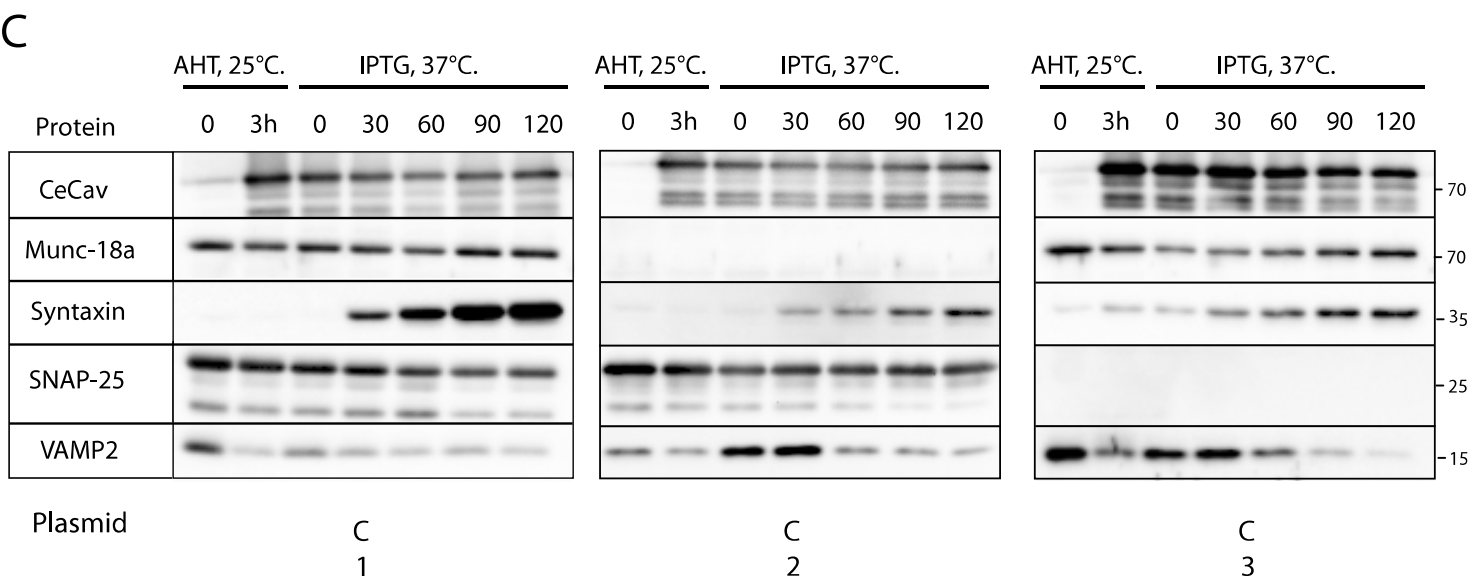

Supplement: FigureS1 [file mmc2.pdf]

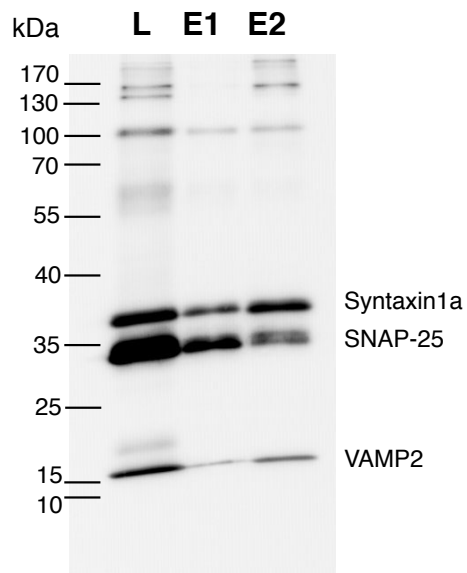

Supplement: FigureS2 [file mmc3.pdf]

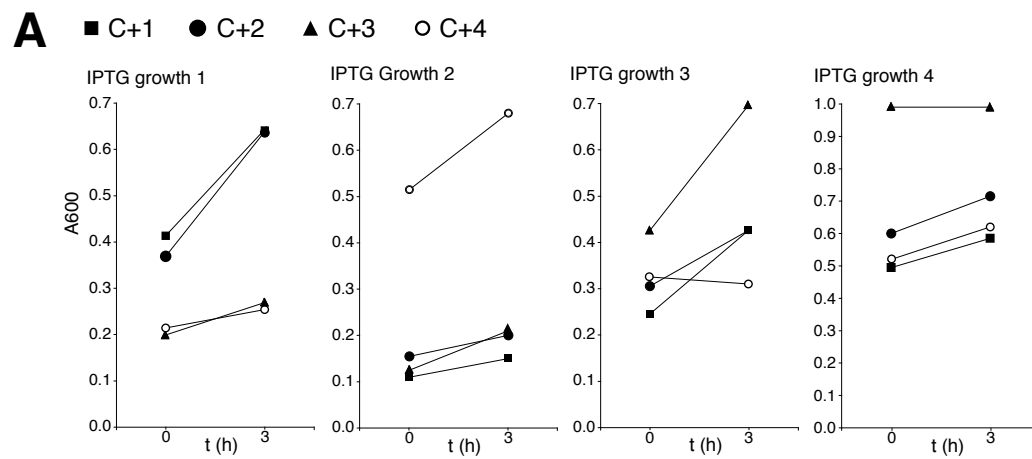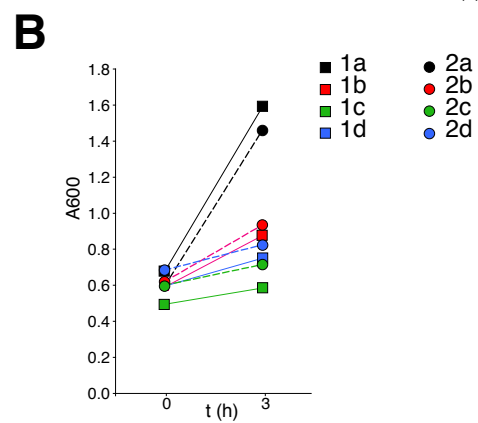

Supplement: FigureS3 [file mmc4.pdf]

A

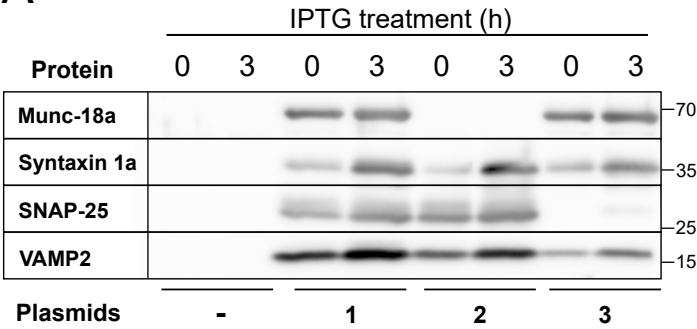

B

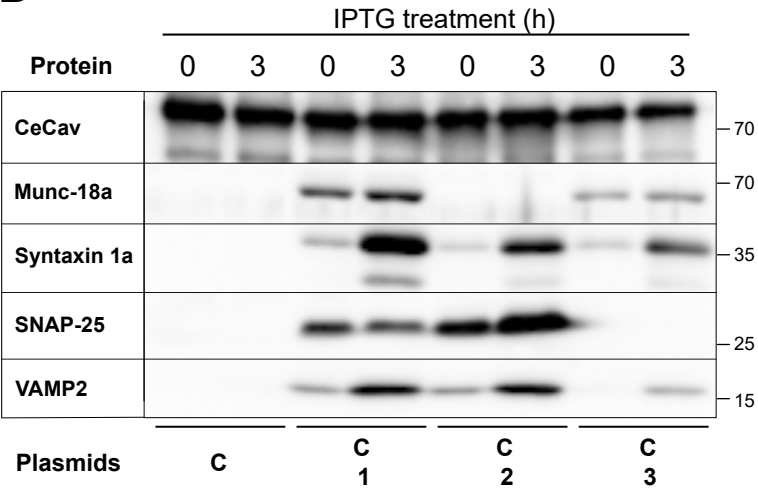

C

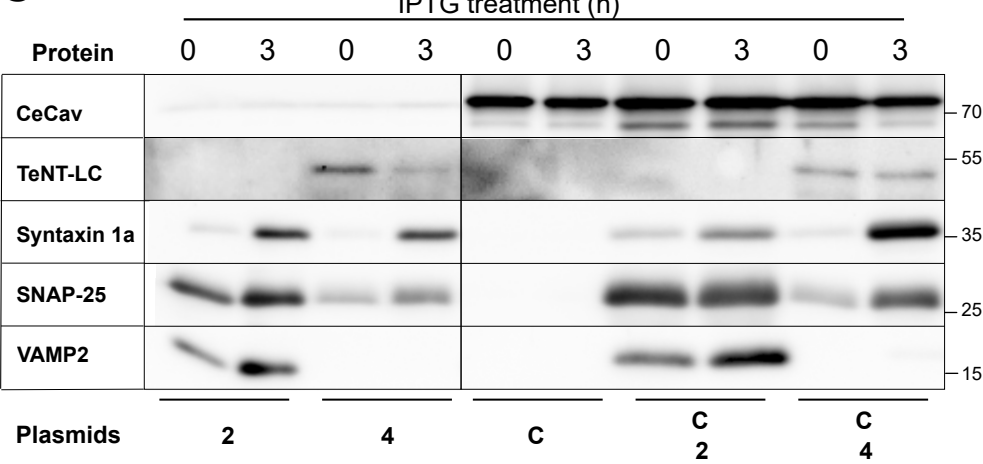

Supplement: FigureS4 [file mmc5.pdf]
